# Supplementary material for: Ketogenic diet restrains aging-induced exacerbation of coronavirus infection in mice
Source: eLife. 2021 Jun 21;10:e66522. doi: 10.7554/eLife.66522 (PMC8245129; doi:10.7554/eLife.66522)
Supplement: Supplementary file 1. — The table includes information about forward and reverse primers for genes used in qPCR experiments. [file elife-66522-supp1.docx]

**Supplementary File 1. Primer information for qPCR**

| **Gene** | **Direction** | **5'-3'** |
| --- | --- | --- |
| *MHV-A59*  *(membrane (M) protein)* | Forward | GGTCAAAGGTTTGGAAGCAG |
|  | Reverse | TGTGAAATGCCACCTTTTGA |
| *Il1b* | Forward | GGTCAAAGGTTTGGAAGCAG |
|  | Reverse | TGTGAAATGCCACCTTTTGA |
| *Tnf* | Forward | TCTCAGCCTCTTCTCATT |
|  | Reverse | AGAACTGATGAGAGGGAG |
| *Il6* | Forward | AGACAAAGCCAGAGTCCTTCAGAG |
|  | Reverse | TTGGTCCTTAGCCACTCCTTCTGT |
| *Casp1* | Forward | GGACCCTCAAGTTTTGCCCT |
|  | Reverse | AGACGTGTACGAGTGGTTGT |
| *Nlrp3* | Forward | GCTAAGAAGGACCAGCCAGA |
|  | Reverse | CAGCAAACCCATCCACTCTT |
| *Gapdh* | Forward | TCAACAGCAACTCCCACTCTTCCA |
|  | Reverse | ACCCTGTTGCTGTAGCCGTATTCA |
